# Contextual Adaptation, Translation, and Validation of the Affiliate Stigma Scale among Primary Caregivers of Children with Physical Disabilities in Nepal: An Observational Study

**DOI:** 10.31729/jnma.9045

**Published:** 2025-06-30

**Authors:** Bibek Banskota, Deepa Bhatta, Rajan Bhusal, Prakash Kumar Yadav, Ashok Kumar Banskota

**Affiliations:** 1 Hospital and Rehabilitation Centre for Disabled Children (HRDC), Banepa, Kavre, Nepal

**Keywords:** *affiliate stigma scale*, *reliability and validity*, *physical disabilities*, *primary caregivers*

## Abstract

**Introduction:**

The experience of affiliate stigma can cause psychosocial problems among caregivers of children with physical disabilities, seriously affecting children's care and rehabilitation. To measure this stigma, we aimed to adapt and validate the widely accepted Affiliate Stigma Scale in the Nepali language.

**Methods:**

This study was conducted among 220 primary caregivers of children with physical disabilities, who presented at the Hospital and Rehabilitation Centre for Disabled Children, Nepal, from April to June 2024. The original tool was adapted, translated, followed by experts' reviews, and pretesting. The final Nepali version was then administered among conveniently selected participants through face-to-face interviews. Data analyses comprised different parts, including item analysis, reliability, validity analysis, and Exploratory Factor Analysis, performed using SPSS version 16.

**Results:**

Item analyses revealed strong item-total correlations (r=0.31-0.76) for all 21 items except A3 (r=0.23). Critical Ratios indicated high discriminative power (CRs>0.30) for all items. The reliability test showed a strong Cronbach's alpha coefficient (0.90), and odd-even split-half reliability (0.83 and 0.80). The tool exhibits good discriminative validity (p=0.04), and high convergent validity (r=-0.33, p<0.00). Moreover, exploratory factor analysis results supported the three-factor structure of the original scale, though some issues were identified: item A3 not loading onto any of the three factors, some cross-loadings, and items from different domains grouping together.

**Conclusions:**

Despite having the problematic structure of the scale, it was still found highly reliable and valid in measuring affiliate stigma among the study population.

## INTRODUCTION

Parenting a child with a disability comes with an additional challenging experience due to the stigma associated with it.^1-3^ Disability is often perceived as an outcome of past sins, karmas, and misfortune, leading to societal stigma and discrimination.^4^ Affiliate stigma in family members, characterized by feelings of stress, guilt, and shame, results in psychological disorders such as depression, anxiety, and poor self-esteem.^5,6^

Though affiliate stigma profoundly impacts caregivers' quality of life, it is one of the least studied issues in Nepal, lacking standard tools for its measurement.^7^ The Affiliate Stigma Scale (ASS), developed by Mak and Cheung,^8^ is widely validated and utilized in different languages to assess the internalized stigma among caregivers of individuals with intellectual disabilities or mental illness.^9-12^

Thus, this study aims to adapt, translate and validate the ASS in the Nepali language among primary caregivers of children with physical disabilities (PDs).

## METHODS

A hospital-based observational cross-sectional study was conducted among primary caregivers of children with PDs presenting to the Hospital and Rehabilitation Center for Disabled Children (HRDC), Nepal, the only pediatric orthopedic hospital in Nepal.^13^ The study was conducted from April to June 2024, after obtaining approval from the Institutional Review Committee of the Baidya & Banskota Hospital (Reference number: B&BIRC-24-06). A total of 220 primary caregivers, specifically parents who provide daily care and support to their children, were conveniently chosen, ensuring 10-15 responses per item, as Pett, Lackey, and Sullivan recommended.^14^ Caregivers of children with treatable acute injuries were excluded. After obtaining informed written consent from each participant, the final Nepali version of the ASS tool was administered through face-to-face interviews using Kobo Collect software. The Nepali version of the Multidimensional Scale of Perceived Social Support (MSPSS-N) was concurrently used to determine the construct validity.^15^

The adaptation and translation process of the tool started after obtaining permission from the original authors. We followed well-accepted and commonly recommended procedures for cross-cultural adaptation and validation of any health measures,^16^ including five stages: 1) contextual modification; 2) multiple forward translations; 3) backward translations; 4) experts' reviews, and 5) pretest.

To adopt the original ASS tool to the issues of children with PDs, the first four researchers reviewed and replaced a few words. For example, in item 1: *I feel inferior because I have a family member with a mental illness',* the word *'family member'* was replaced with 'child', *and 'mental illness'* was replaced with *'physical disability'.* The modified version (Draft 1) was then proceeded for translations. Two native bilingual translators from medical and non-medical backgrounds independently translated the original English version into the Nepali language (Draft 2 and Draft 3). One of the translators was a public health researcher with more than 10 years of experience. The second translator was a non-medical professional with an extensive understanding of Nepali culture. Synthesizing these two translations, the research team, along with two translators, produced a unified Draft 4. The reverse translation of Draft 4 was performed by a bilingual senior orthopedic surgeon who was unfamiliar with the research concepts and ASS tool. The back-translated version was named Draft 5.

The expert team, including all three translators, two orthopedic surgeons, and three researchers, reviewed and consolidated all the translations into Draft 6. The term *'behavior'* in item 3: *'The behavior of my family member with mental illness makes me feel embarrassed'* did not relate to the concept of physical disability, and was replaced with *'condition'* through consensus.

The pre-final Nepali version (Draft 6) was then pretested among ten similar participants. Face-to-face interviews were conducted with each participant to assess the clarity and understandability of the translated tool. An unclear and misinterpreted phrase, *'makes me lose face'* in item 14: *'Having a family member with mental illness makes me lose face'* was simplified to *'makes me lose respect'.* Apart from these, none of the items were found unclear, and researchers reached a final consensus (ASS-N), which is provided in the Supplementary File (Supplementary File 1).

The variables included were age, sex, ethnicity, educational status, parenting status, and period of physical disabilities. The major ethnic groups included were Brahmin/Chhetri, Janajati, Dalit, Madhesi, and Muslim.^17^ Data were collected using the Kobo Collect Software and exported to the IBM SPSS version 16 for the analysis. The item-total correlation (ITC) and corrected-item-total correlation (CITC) were analyzed to determine the homogeneity of items. Items with a correlation coefficient ≥0.30 were considered appropriate.^9^ A significant (p<0.05) independent t-test between the top 27% (highest 59 cases) and the bottom 27% (lowest 59 cases) groups indicated good discriminative power of the tool. Additionally, items with Critical Ratios (CRs) higher than 3.00 were identified as strongly discriminatory.^18^ Cronbach's alphas if-item deleted, within each domain: Affective, Cognitive, Behavioral, and odd-even split-half reliabilities were calculated to evaluate the retention of each item in further analyses. It was suggested to remove the item from the scale if Cronbach's alpha coefficient <0.70.

To assess concurrent validity, a bivariate correlation analysis was performed between ASS-N and MSPSS-N, expecting a negative correlation.^19^ Finally, to determine the factor structure of ASS-N, EFA was performed using the principal component analysis (PCA) and varimax rotation. Kaiser-Meyer-Olkin (KMO) was used to test the strength of association among the variables, and Bartlett's test for sphericity was used to assess the overall significance of all the correlations within the correlation matrix. The data set was considered appropriate for factor analyses if both the Bartlett test of sphericity (P<0.05) and the KMO were significant (KM0>0.60). The number of factors to be extracted was determined by combining two methods: the eigenvalues-greater-than-one-rule, and the Monte Carlo PCA for parallel analysis.^20,21^ As recommended by Hayton et al.^22^, the intersection point of eigenvalues in our data and eigenvalues of a group of random matrices was identified. Items with communalities higher than 0.20 or higher and items with factor loadings >0.40 were considered acceptable for the scale.^23^

## RESULTS

Among 220 participants, most participants, 139 (63.18%), were mothers, with age of 32.71 ±5.88 years. The Brahmin/Chhetri ethnic group comprised 89 participants (40.45%), followed by 48 Janajati (21.81%), and 40 Madhesi (18.18%). Out of 220, 77 participants (35.00%) reported completing secondary- level education, and 205 (93.18%) participants were rearing their children with co-parenting. Among the total sample, children of 125 (56.82%) participants had been living with disability for more than five years (Table 1).

**Figure 1:**
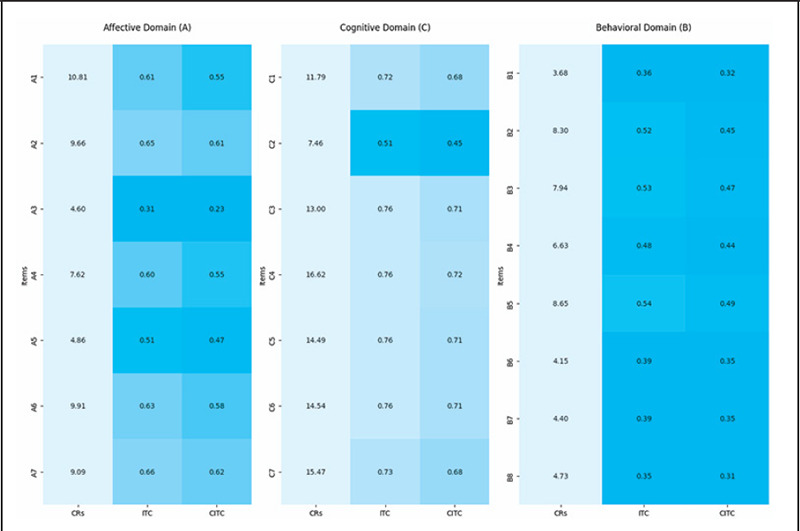
Critical ratios, item-total, and corrected item-total correlations (n=220). CRs: Critical Ratios; ITC=Item Total Correlation; CITC=Corrected Item-Total Correlation; A: Affective; C: Cognitive; B: Behavioral.

The results of item analyses revealed that ITC ranged from 0.31 to 0.76 while CITC ranged from 0.23 to 0.72; however, for A3, CITC was 0.23. Additionally, discriminative power, which is CR, ranged from 4.15 to 16.62 (Figure 1).

**Table 1:** Baseline characteristics of the primary caregivers of children with physical Disabilities (n=220).

Characteristics	n(%)
**Participants**	
Mother	139(63.18)
Father	81(36.81)
**Ethnicity**	
Brahmin/Chhetri	89(40.45)
Janajati	48(21.81)
Dalit	33(15.00)
Madhesi	40(18.18)
Muslim	10(4.54)
**Educational Status**	
No formal Education	51(23.81)
Primary Level	72(32.72)
Secondary Level	77(35.00)
Higher Education	20(9.09)
**Parenting**	
Co-parenting	205(93.18)
Single parenting	15(6.81)
**Periods of physical disability**	
≤ 1 year	28(12.73)
1 to 5 years	67(30.45)
>5 years	125(56.82)

The Cronbach's alpha value for the entire scale was 0.91, and for each of the affective, cognitive, and behavior domains was 0.77, 0.90, and 0.86, respectively. The split-half reliability for part one was 0.83, and for part two it was 0.80 (Table 2).

**Table 2:** Item-wise internal consistencies (n=220).

ASS Item	Cronbach's alpha if-item deleted
Affective Domain	
A1	0.9
A2	0.9
A3	0.91
A4	0.9
A5	0.9
A6	0.9
A7	0.9
Cognitive Domaine	
C1	0.9
C2	0.9
C3	0.9
C4	0.9
C5	0.9
C6	0.9
C7	0.9
Behavioral Domain	
B1	0.9
B2	0.9
B3	0.9
B4	0.9
B5	0.9
B6	0.9
B7	0.9
B8	0.9

**Figure 2:**
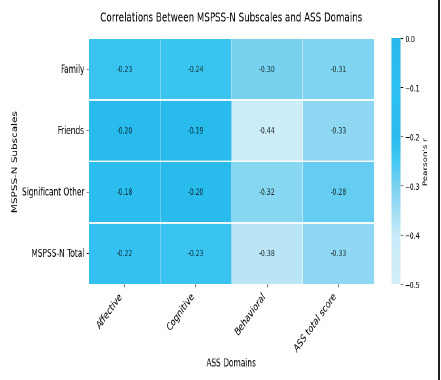
Correlation analysis between MSPSS-N and ASS scores (n=220). ASS=Affiliate Stigma Scale; MSPSS-N =Multidimensional Scale of Perceived Social Support

There was convergent validity, and a negative correlation between the ASS-N and MSPSS-N scores (r=-0.33, p<0.00) (Figure 2).

In the EFA, the KMO measure was 0.88 (>0.60), and the Bartlett test of sphericity was significant (x2 = 3182.15; p=0.00). We fixed the number of factors to be extracted at three, as observed, the plot of five factors from the EFA intersected with the plot of 10 factors from the parallel analysis at three. The varimax rotation results showed 34.66%, 16.62%, and 6.63% of variance. The communalities of all items ranged from 0.26 to 0.81 (>0.20) (Table 3).

Factor loadings ranged from 0.54 to 0.80, all exceeding 0.40. However, item A3 from the affective domain *'The condition of my child with physical disabilities makes me feel embarrassed'* did not load onto any factors. All items of the affective domain loaded onto the 1^st^ factor with factor loadings ranging from 0.54 to 0.70, while item A1 loaded onto the 3^rd^ factor. Moreover, five items of the cognitive domain loaded onto the 1^st^ factor (factor loadings: 0.63-0.80) alongside the items of the affective domain. Meanwhile, items C5 and C6 from the cognitive domain loaded with item A1 from the affective domain to construct 3^rd^ factor. Also, item C6 showed cross-loadings, with a higher loading (0.74) on the 3^rd^ factor together with items A1 and C5. Besides these findings, all the items from the behavioral domain did not load onto 3^rd^ factor but rather loaded together in the 2^nd^ factor with factor loadings ranging from 0.62 to 0.80 (Table 3).

**Table 3:** Summary of factor loadings and communalities for EFA (n=220).

Items	Factor loadings			Communalities
	1	2	3	
A2	0.54			0.50
A4	0.56			0.47
A5	0.64			0.45
A6	0.76			0.59
A7	0.70			0.54
C1	0.80^c^			0.68
C2	0.69^c^			0.48
C3	0.80^c^			0.70
C4	0.74^c^			0.65
C7	0.63^c^			0.60
B1		0.64^d^		0.41
B2		0.66^d^		0.59
B3		0.65^d^		0.54
B4		0.80^d^		0.66
B5		0.75^d^		0.61
B6		0.78^d^		0.61
B7		0.80^d^		0.66
B8		0.62^d^		0.48
C5			0.75^e^	0.81
C6	0.50^f^		0.74^e^	0.81
A1			0.73^a^	0.64
A3^b^			0.26

a Item A^1^ from affective domain did not load into the 1^st^ factor but into the 3^rd^ factor

b Item A^3^ did not show loading on any of the three factors and with borderline communality

c Items from the cognitive domain loaded on 1^st^ factor along with items from the affective domain

d All the 8 items from the behavioral domain did not load into the 3^rd^ factor but rather loaded into the 2^nd^ factor

e The original cognitive domain was split, where items C5 and C6 were loaded together into 3^rd^ factor

f Item C6 had cross-loadings into the 1^st^ and 3^rd^ with the higher value in 3^rd^ factor

## DISCUSSION

The results of item analyses revealed that ITC ranged from 0.31 to 0.76 (p<0.05), while CITC ranged from 0.23 to 0.72 (≥0.30), except the A3 (CITC: 0.23). The critical ratios for all items range from 4.15 to 16.62. The Cronbach's alpha value for the entire scale was 0.91, with all its domains having values more than 0.7 (affective: 0.77; cognitive: 0.90; and behavior: 0.86). The EFA shows the KMO measure as 0.88, with a significant Bartlett test. The three-factor structure was fixed with factor loadings ranging from 0.54 to 0.80. Item A3 had no loadings on any factors, while A1 loaded onto the 3^rd^ factor along with C5 and C6. Five items from the cognitive domain loaded onto the 1^st^ factor alongside the items of the affective domain. Item C6 had crossloadings. All the items from the behavioral domain loaded onto the 2^nd^ factor.

The positive item-total correlations and more than 0.30 critical ratios in the present study are in line with previous studies from Persia (ITC method) and Indonesia (ITC and CITC methods), which showed strong item correlations.^11,24^ In contrast, studies from India, Malaysia, and Greece did not report using any of these methods.^11,12,25^ The Cronbach's alpha of more than 0.70 in the present study is consistent with the results from previous studies, though none of the studies reports assessing split-half reliability.^9,11,25^

The identified three-factor structure of the tool in this study aligns with the original scale developed by Winnie W.S. and Rebecca Y.M.^8^ The number of factors extracted aligns with the previous studies from different countries, however, the psychometric evaluation of the ASS in those studies differs by methods, and samples used.^9,11,12,25^ Only one study from Turkey has been found to include a sample of spinal cord injury/ disorder caregivers, however, the methods used slightly differed from the present study.^10^ Regardless of the context, the measure of KMO sampling adequacy was high (0.88) with a significant Bartlett's test result, which aligns with the findings from the previous studies from India and Malaysia (KMO=0.90, 0.92, respectively, and a significant Bartlets's test.^12,25^ Furthermore, it is important to note some differences regarding the scale structure between the original scale and the results of the present study. In our study, the items from the affective and cognitive domains clustered together to form one factor. This might be expected because items from both domains can be intertwined, as theorized in the integrated cognitive- affective model.^26^ Although most items of the affective and cognitive domains were not strongly correlated in our sample ('r' ranging from 0.14 to 0.52), the factor analysis did not discriminate between these two constructs. Moreover, two items (C5 and C6) from the cognitive domain loaded together with the A1 item from the affective domain to form the 3^rd^ factor. One of the possible explanations could be that all three items measure the same experience of decreased self-esteem—feeling inferior, lesser, and incompetent than others. Item C6 showed cross-loading (0.50) onto the first clustered group of affective and cognitive domains, but loading onto the 3^rd^ factor was high (0.74). Interestingly, item A3 (measuring embarrassment or shame) from the affective domain did not load onto any of the three factors in our sample. This might be attributed to the evidence that people can perceive PDs as merely a physical condition, not a matter of shame.^19,27^ Therefore, item A3 might not be feasible in the context of physical disability. Furthermore, items from the behavior domain did not load onto their assigned scale but loaded together onto the 2^nd^ factor in our solution. Therefore, we can suggest that factor two can be renamed as a behavioral domain.

Moreover, there was a significant negative correlation, as expected ^19,24^ between the obtained scores of ASS-N and the MSPSS-N ('r' ranging from -0.18 to -0.44), suggesting that parents with lower social support experience higher levels of affiliate stigma.

Some differences in our factor analyses are not unexpected, since we tested the original tool on a different sample. Therefore, results cannot be used as a whole basis for changing the structure of the original scale. We recommend further exploration and fine- tuning of the tool, focusing on item A3 of the affective domain, which showed borderline communality and did not load onto any of the three domains in our analysis. Nevertheless, replicating the original tool within the context of PDs exhibits a significant scientific contribution. Ensuring 10 to 15 responses per item, we included 220 participants, enough to stabilize the factor structure of the tool.^28,29^ However, a few limitations are important to note. First, our study is limited to the parents of children with PDs and might not apply to caregivers other than family caregivers. Secondly, data collection was confined to a single facility using a convenient sampling technique. However, being the only pediatric orthopedic hospital in Nepal and serving children from all regions of the nation ^13^, we believe that the findings can be generalized for Nepal.

## CONCLUSIONS

Though there are some issues with the factor structure, the adapted and translated scale (ASS-N) was found to be highly reliable and valid in our sample. All the items are equally important in measuring the broader construct of internalized stigma. However, we recommend further exploration and fine-tuning of the tool, focusing on item A3 of the affective domain, which showed borderline communality and did not load onto any of the three domains in our analysis.
